# Postoperative adjuvant immunotherapy for pathological stage II–IVa esophageal squamous cell carcinoma after radical surgery does not improve disease-free recurrence rates

**DOI:** 10.3389/fmed.2024.1517001

**Published:** 2024-12-13

**Authors:** Xihao Xie, Hai Zhang, Haiquan He, Bomeng Wu, Ying Chen, Wanli Lin, Qingyi Feng, Qunqing Chen

**Affiliations:** ^1^Department of Thoracic Surgery, Zhujiang Hospital, Southern Medical University, Guangzhou, China; ^2^Department of Thoracic Surgery, Gaozhou People’s Hospital Affiliated to Guangdong Medical University, Gaozhou, China

**Keywords:** adjuvant, chemotherapy, esophageal cancer, immunotherapy, disease-free recurrence rates

## Abstract

**Background/objectives:**

Postoperative adjuvant therapy for esophageal squamous cell carcinoma (ESCC) primarily includes chemotherapy and chemoradiotherapy. The survival benefits of postoperative adjuvant therapy for R0-resected ESCC remain controversial. Immunotherapy is being gradually applied perioperatively for esophageal cancer, but the efficacy of postoperative immunotherapy in ESCC is unclear. This study aimed to evaluate the effectiveness of postoperative immunotherapy for esophageal cancer. Toward this goal, we explored the differences between postoperative immunotherapy combined with chemotherapy and postoperative adjuvant chemotherapy alone.

**Methods:**

This retrospective study evaluated patients who underwent radical surgery for esophageal cancer at Gaozhou People’s Hospital between January 2020 and August 2022 and received postoperative adjuvant therapy. Patients were divided into two groups according to the adjuvant treatment regimens: postoperative adjuvant chemotherapy (aCT) and postoperative adjuvant immunotherapy combined with chemotherapy (aICT) groups. Data on baseline characteristics, surgical-related indicators, adverse event rates during adjuvant therapy, and 2-year postoperative follow-up were collected for both groups.

**Results:**

A total of 76 patients were included: 36 and 40 patients in the aICT and aCT groups, respectively. There were no significant differences in baseline data between the two groups. During the adjuvant treatment period, the incidence of hypothyroidism was significantly higher in the aICT group than in the aCT group (25.0% vs. 2.5%, *p* = 0.007). During the 2-year follow-up, local and recurrence rates were 17.5 and 12.5% in the aCT group and 13.9 and 5.6% in the aICT group, respectively, showing no significant difference between the two groups (*p* = 0.489).

**Conclusion:**

For patients with pathologically confirmed locally advanced ESCC after surgery, postoperative immunotherapy did not confer better disease-free recurrence rates compared to postoperative adjuvant therapy. Nonetheless, with research advancements, the role of immunotherapy in the treatment of ESCC is likely to expand, offering new hope for these patients.

## 1 Introduction

Surgery is the primary treatment modality for early- and mid-stage esophageal squamous cell carcinoma (ESCC). Surgical techniques have rapidly advanced over the past decade, with new technologies being increasingly applied in clinical practice, particularly in minimally invasive treatments and robotic surgeries for esophageal cancer (1). These advancements and innovations have significantly improved the patients’ quality of life; however, long-term efficacy remains less than satisfactory (2, 3). Current research indicates that patients may still experience local recurrence or distant metastasis within 2 years after radical surgery for esophageal cancer. This directly affects their survival prognosis, with a 5-year survival rate of approximately 25–30% (4–6). Particularly, postoperative pathological findings indicating positive lymph nodes directly influence patient survival outcomes (7). To further improve long-term survival rates, perioperative neoadjuvant and adjuvant therapies for locally advanced esophageal cancer have become key research focuses (8, 9). Meta-analyses show that neoadjuvant chemoradiotherapy or neoadjuvant chemotherapy is beneficial in improving survival outcomes for patients with locally advanced esophageal cancer. In clinical practice, it is common to combine neoadjuvant treatment with surgery, combine surgery with adjuvant treatment, or implement a comprehensive treatment approach that includes perioperative adjuvant therapies (neoadjuvant treatment, surgery, and postoperative adjuvant treatment), although specific protocols may vary across different clinical centers (10–12).

Neoadjuvant therapy encompasses neoadjuvant chemoradiotherapy, neoadjuvant chemotherapy, and, increasingly, neoadjuvant immunotherapy adopted in clinical trials. These therapeutic approaches have been shown to enhance R0 resection rates, prolong disease-free survival, and potentially improve long-term survival rates (13, 14). Postoperative adjuvant therapy is routinely offered as a complementary treatment to mitigate the risk of postoperative recurrence and improve the survival of esophageal cancer patients with postoperative pathological stages of T2 or higher, N+, or other high-risk factors. Postoperative adjuvant chemotherapy is the most common adjuvant regimen, although some institutions also perform postoperative chemoradiotherapy (15). However, there exists some controversy regarding the ability of postoperative adjuvant therapy to improve long-term survival for esophageal cancer. For select patients with R0-resected T3, N+ ESCC, postoperative adjuvant radiotherapy can reduce postoperative recurrence and improve survival (16). Conversely, postoperative adjuvant chemotherapy may be a risk factor for poor disease-free survival (DFS) and overall survival (OS) (17). Immunotherapy has demonstrated promising efficacy in the treatment of advanced esophageal cancer, improving survival outcomes. Consequently, the exploratory application of immunotherapy in the perioperative period of esophageal cancer has become increasingly prevalent (18). Researchers are actively investigating whether immunotherapy can effectively identify and attack cancer cells in postoperative patients, thereby providing these patients with more comprehensive and durable therapeutic effects (19). However, there are fewer studies on the safety and efficacy of the novel treatment paradigm of postoperative adjuvant immunotherapy compared to those on traditional postoperative adjuvant chemotherapy. Hence, this study aimed to assess the real-world short-term efficacy of immunotherapy in the adjuvant setting for postoperative patients with ESCC.

## 2 Methods

### 2.1 Study design and patients

This retrospective study was approved by the Ethical Committee of Gaozhou People’s Hospital and was conducted according to the tenets of the Declaration of Helsinki. The requirement for individual consent was waived.

Patients with esophageal cancer who underwent surgical resection and postoperative adjuvant therapy at Gaozhou People’s Hospital between January 2020 and August 2022 were evaluated. The inclusion criteria were as follows: (1) age 18–75 years; (2) underwent minimally invasive Mckeown surgery; (3) postoperative pathological confirmation of ESCC; (4) postoperative pathological stage I–IVa ESCC; (5) received postoperative adjuvant chemotherapy or immune therapy combined with chemotherapy; and (6) complete clinical data. The exclusion criteria were as follows: (1) preoperative severe cardiovascular and cerebrovascular complications; (2) preoperative coexistence of other malignancies; (3) preoperative neoadjuvant therapy; (4) non-R0 resection of esophageal cancer; and (5) postoperative adjuvant radiotherapy. The patients were divided into two groups based on the adjuvant treatment regimen received: postoperative adjuvant chemotherapy (aCT) or postoperative adjuvant immunotherapy combined with chemotherapy (aICT).

### 2.2 Postoperative adjuvant treatment regimens

#### 2.2.1 Postoperative adjuvant chemotherapy

Postoperative adjuvant chemotherapy was administered every 3 weeks for a total of 4 cycles. Chemotherapy drugs included docetaxel (75 mg/m^2^), paclitaxel injection (135 mg/m^2^), paclitaxel injection (albumin bound, 260 mg/m^2^), fluorouracil injection (0.5–1 g, d1–5), and vinorelbine bitartrate injection (25–30 mg/m^2^, d1 and d8) in combination with cisplatin (75 mg/m^2^) or carboplatin injection (200–400 mg/m^2^).

#### 2.2.2 Postoperative adjuvant chemotherapy combined with immunotherapy

Postoperative adjuvant chemotherapy combined with immunotherapy was administered every 3 weeks for a total of 4 cycles. The chemotherapy regimen was the same as that in the postoperative adjuvant chemotherapy group. The immunotherapy drugs included sintilimab injection (200 mg every 3 weeks), tislelizumab injection (200 mg every 3 weeks), camrelizumab injection (200 mg every 3 weeks), toripalimab (3 mg/kg, every 2 weeks), pembrolizumab injection (2 mg/kg every 2 weeks), or nivolumab injection (3 mg/kg every 2 weeks).

### 2.3 Observation indicators

Patient data, including demographic information, past medical history, personal history, tumor pathological stage, tumor location, surgery-related indicators (operative time, intraoperative blood loss, number of lymph node stations dissected, number of lymph nodes dissected, postoperative hospital stay, and postoperative complication rate), incidence of adverse events during postoperative adjuvant treatment, tumor recurrence pattern at 2 years after surgery, and recurrence-free survival time at 2 years after surgery, were collected. Postoperative pathological staging was based on the 8th edition of the American Joint Committee on Cancer/Union for International Cancer Control staging of cancers of the esophagus and esophagogastric junction. The incidence of adverse events during postoperative adjuvant treatment was assessed using the Common Terminology Criteria for Adverse Events version 5.0 Local recurrence and metastasis was defined as those involving the mediastinal lymph node, supraclavicular lymph node, and local anastomotic recurrence. However, distant metastasis was defined as metastasis to distant organs.

### 2.4 Statistical analysis

Age, operative time, intraoperative blood loss, number of lymph node dissection, number of lymph node dissection stations, and postoperative hospitalization days were expressed as the mean ± standard deviation and analyzed using Student’s t-test. Sex, combined history of hypertension and diabetes, tumor location, postoperative pathological G-grade, postoperative pathological tumor stage, incidence of postoperative anastomotic fistula, pulmonary infections, myelosuppression during postoperative adjuvant therapy, liver function impairment, hypothyroidism, and diarrhea were analyzed. The rates of local recurrence and distant metastasis at 2 years after surgery were assessed using the Chi-square test. The DFS was calculated using the Kaplan–Meier method, and differences in relapse-free survival rates were evaluated with the log-rank test. Confidence intervals (CIs) were set at 95%. All statistical analyses were performed using SPSS (Version 23; IBM, Armonk, NY, USA) and GraphPad Prism version 9.0 software (GraphPad Software, La Jolla, CA, USA). A two-sided *p*-value <0.05 was considered statistically significant.

## 3 Results

**Figure 1:**
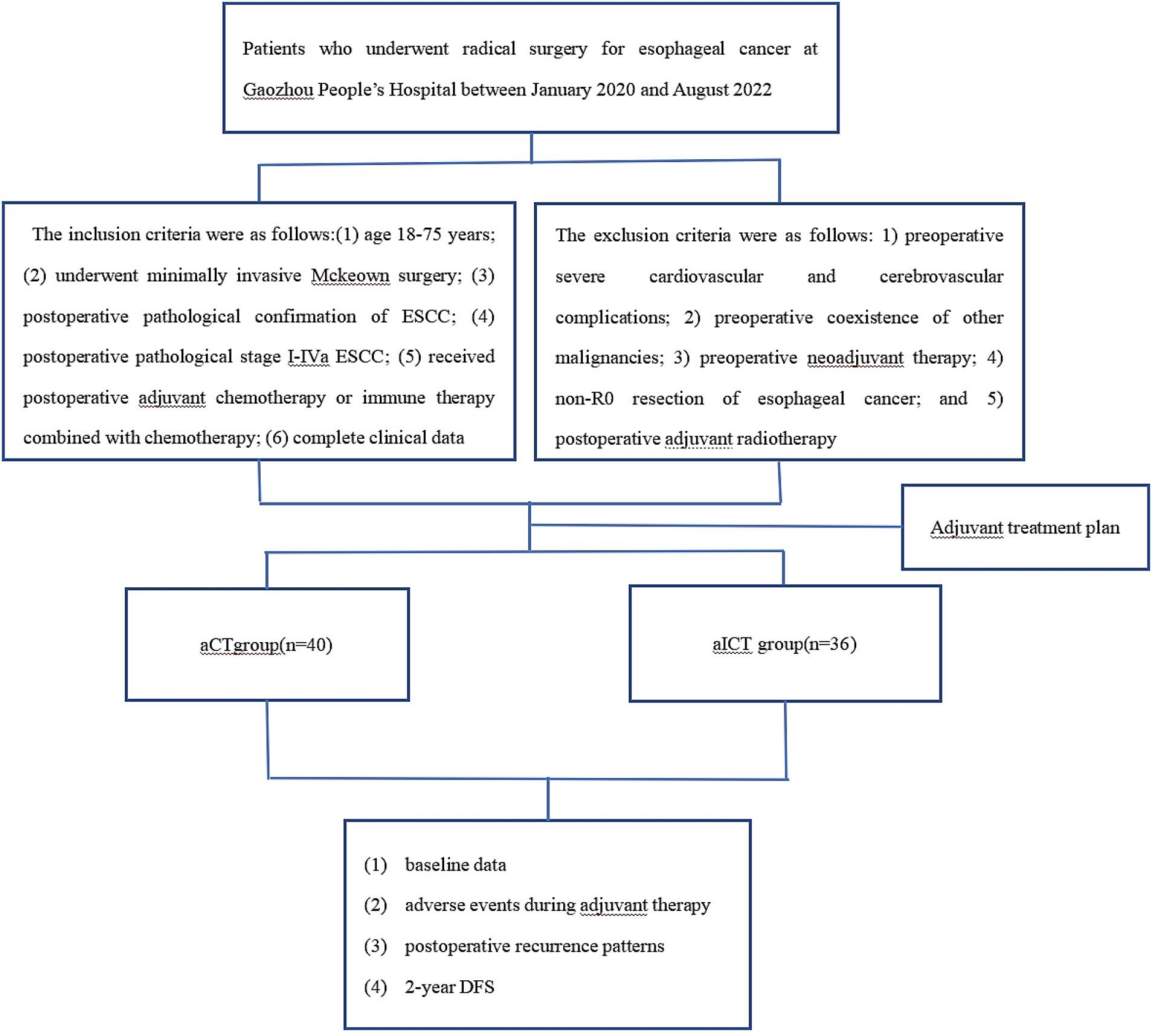
Study flowchart. aCT, adjuvant chemotherapy; aICT, adjuvant immunotherapy combined with chemotherapy; DFS, disease-free survival.

**Figure 2:**
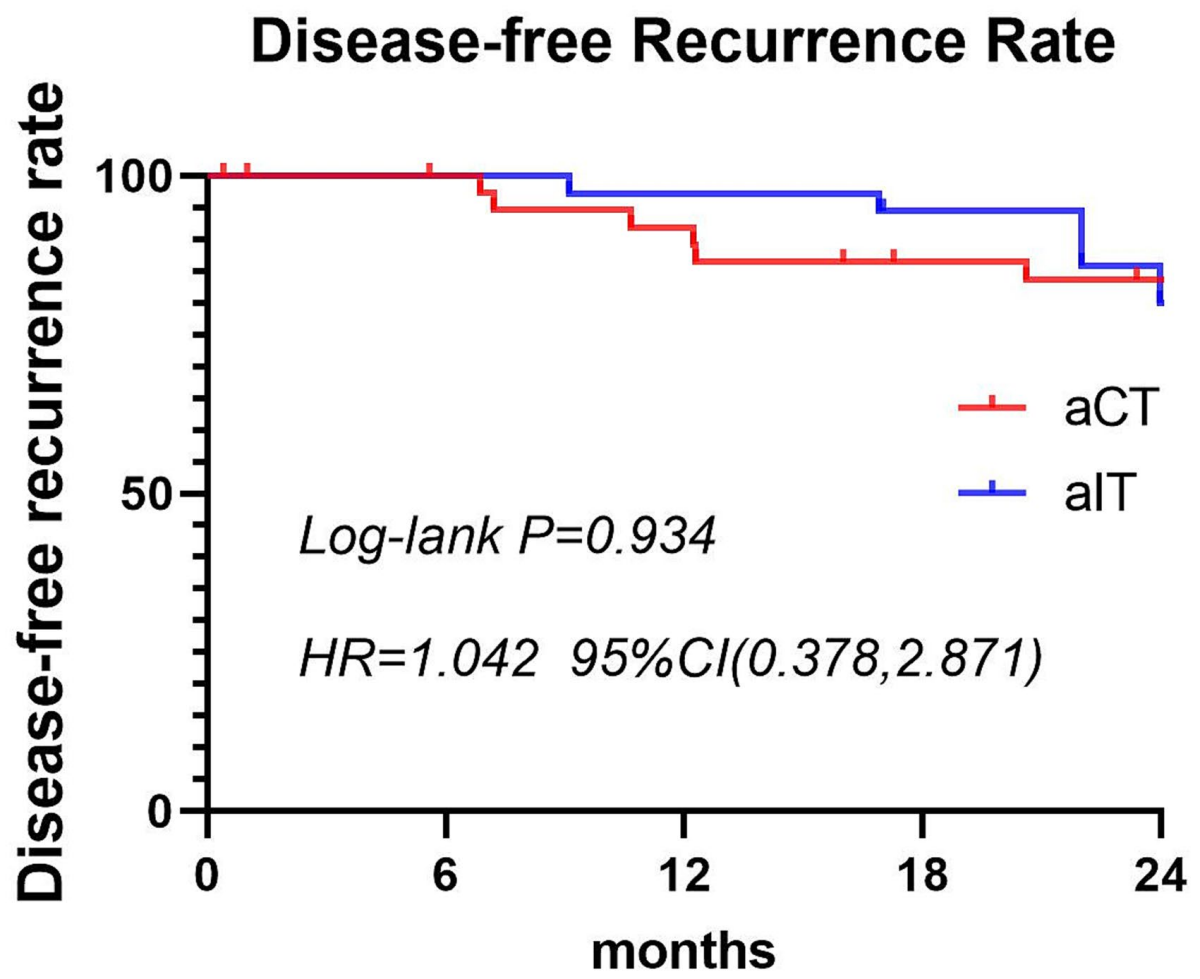
Comparison of 2-year disease-free survival between the two groups of patients after surgery. aCT, adjuvant chemotherapy; aICT, adjuvant immunotherapy combined with chemotherapy; DFS, disease-free survival.

A total of 76 patients were included; among them, 40 and 36 patients belonged to the aCT and aICT groups, respectively. The study flowchart is shown in Figure 1. The aICT group was older but the difference in age was not significant (mean: 61.850 ± 7.973 years vs. 65.194 ± 8.558 years, *p* > 0.05). There were also no significant between-group differences in sex, postoperative pathological G grading, pathological staging, perioperative surgical-related indicators, and the incidence of postoperative complications. The patient characteristics are shown in Table 1. The main complications during the adjuvant treatment period included bone marrow suppression, elevated liver transaminases, hypothyroidism, and diarrhea. The incidence rates of hypothyroidism were 2.5% (*n* = 1, grade 1) in the aCT group and 25% in the aICT group (*n* = 9, 2 [5.6%] patients with grade 2 hypothyroidism), with a significant difference (*p* < 0.05, Table 2). The median follow-up time for the overall population was 28.5 months. The rates of local recurrence and distant metastasis were higher in the aCT than in the aICT group, although the difference was not significant (Table 3). There was also no significant between-group difference in DFS (HR = 1.042, 95% CI = 0.378–2.871, *p* = 0.934). The survival curves for both groups are shown in Figure 2.

**Table 1:** Comparison of baseline data between the two groups.

Clinical features	aCT group (*n* = 40)	aICT group (*n* = 36)	*χ*^2^/*t*	*p*
Age	61.850 ± 7.973	65.194 ± 8.558	−1.764	0.082
Sex (%)			0.045	0.831
Male	28 (70.0)	25 (72.2)		
Female	12 (30.0)	11 (27.8)		
History of hypertension (%)	4 (10.0)	3 (8.3)	0.062	0.802
History of diabetes (%)	1 (2.5)	1 (2.8)	0.006	0.940
Tumor location (%)			1.935	0.380
Upper	3 (7.5)	3 (8.3)		
Middle	13 (32.5)	17 (47.2)		
Lower	24 (60.0)	16 (44.4)		
G stage (%)			0.381	0.827
G_1_	2 (5.0)	3 (8.3)		
G_2_	21 (52.5)	19 (52.8)		
G_3_	17 (42.5)	14 (38.9)		
Pathological stage (%)			0.663	0.718
II	9 (22.5)	6 (16.7)		
III	29 (72.5)	27 (75.0)		
IV	2 (5.0)	3 (8.3)		
Operation time (min)	288.475 ± 57.751	302.722 ± 59.100	−1.062	0.292
Intraoperative blood loss (mL)	88.000 ± 28.029	94.444 ± 35.411	−0.884	0.380
Number of lymph nodes dissected	28.825 ± 7.382	31.277 ± 12.427	−1.058	0.293
Number of lymph node dissection stations	11.625 ± 1.705	12.416 ± 2.622	−1.575	0.119
Postoperative hospital stay	12.250 ± 5.623	13.388 ± 10.340	−0.605	0.547
Postoperative pulmonary infection (%)	2 (5.0)	3 (8.3)	0.015	0.903
Anastomotic fistula (%)	2 (5.0)	1 (2.8)	2.47	0.619

Data are presented as *n* (%) or mean ± SD.

aCT, adjuvant chemotherapy; aICT, adjuvant immunotherapy combined with chemotherapy.

**Table 2:** Comparison of the incidence of adverse events during adjuvant therapy after surgery between the two groups of patients.

Variables	aCT group (*n* = 40)	aICT group (*n* = 36)	*Z*	*p*
Myelosuppression (%)			1.566	0.815
Grade 0	25 (62.5)	21 (58.3)		
Grade 1	5 (12.5)	7 (19.4)		
Grade 2	6 (15.0)	5 (13.9)		
Grade 3	3 (7.5)	3 (8.3)		
Grade 4	1 (2.5)	0 (0.0)		
Glutamic pyruvic transaminase increased (%)			1.134	0.567
Grade 0	31 (77.5)	24 (66.7)		
Grade 1	7 (17.5)	9 (25.0)		
Grade 2	2 (5.0)	3 (8.3)		
Glutamic oxaloacetic transaminase increased (%)			0.086	0.958
Grade 0	29 (72.5)	25 (69.4)		
Grade 1	8 (20.0)	8 (22.2)		
Grade 2	3 (7.5)	3 (8.3)		
Hypothyroidism (%)			9.818	0.007
Grade 0	39 (97.5)	27 (75.0)		
Grade 1	1 (2.5)	7 (19.4)		
Grade 2	0 (0.0)	2 (5.6)		
Diarrhea (%)			0.773	0.379
Grade 0	37 (92.5)	30 (83.3)		
Grade1	3 (7.5)	6 (16.7)		

aCT, adjuvant chemotherapy; aICT, adjuvant immunotherapy combined with chemotherapy.

**Table 3:** Comparison of postoperative recurrence patterns between the two groups.

Variables	aCT group (*n* = 40)	aICT group (*n* = 36)	*Z*	*p*
Tumor recurrence (%)			1.430	0.489
Mediastinal lymph nodes/esophagus	7 (17.5)	5 (13.9)		
Distant metastasis	5 (12.5)	2 (5.6)		

aCT, adjuvant chemotherapy; aICT, adjuvant immunotherapy combined with chemotherapy.

## 4 Discussion

The introduction of immune checkpoint inhibitors in clinical practice has led to notable advancements in the treatment of esophageal cancer. The KEYNOTE-590 study demonstrated that the first-line administration of immunotherapy significantly enhanced long-term survival rates (20). Furthermore, a meta-analysis confirmed the efficacy of immunotherapy as a first-line treatment for advanced esophageal cancer (21). The success of immunotherapy as first-line treatment for advanced esophageal cancer has prompted the focus on its clinical application in the perioperative setting. However, the controversy continues. Nevo et al. (22) reported negative outcomes associated with the combination of neoadjuvant immunotherapy and chemotherapy. In contrast, Zhang et al. (23) found that neoadjuvant immunotherapy may provide significant survival benefits for patients with resectable locally advanced ESCC. Postoperative adjuvant therapy is currently not a standard treatment modality for resectable esophageal cancer. There is relatively fewer research data on postoperative adjuvant immunotherapy than on neoadjuvant immunotherapy. There are ongoing clinical trials exploring the role of immune checkpoint inhibitors in postoperative adjuvant treatment, either alone or in combination with other therapies, in solid tumors (24). Given the high recurrence rate of esophageal cancer, there is hope for more clinical studies to further explore this new treatment model of postoperative adjuvant immunotherapy for esophageal cancer (25–27).

Immunotherapy can activate the patient’s immune system and enhance its ability to attack cancer cells, but it also has side effects. Adjuvant therapy has clinical specificity, particularly for patients who have undergone R0 surgical resection, as the patient’s physical condition postoperatively is worse than that preoperatively. In the current study, the addition of immunotherapy agents did not significantly increase the incidence of common hematological and biochemical complications (e.g., as bone marrow suppression and liver dysfunction) during the adjuvant chemotherapy period. However, the incidence of hypothyroidism during the postoperative immunotherapy period was higher in those receiving postoperative adjuvant immunotherapy combined with chemotherapy than in those receiving adjuvant chemotherapy alone, although this was limited to grade 1 hypothyroidism. For other common immune-related adverse events, no significant increase was found in this study, which may be due to the short postoperative immunotherapy cycle and the small study sample size. In the CheckMate 577 study, the incidence of grade ≥ 3 adverse events was less than 1% in the postoperative maintenance immunotherapy group (25). In a prospective clinical trial of patients with locally advanced esophageal cancer conducted by Park et al. (27), the immunotherapy-related complications following durvalumab maintenance after neoadjuvant chemoradiotherapy was largely limited to grades 1 and 2. Collectively, these findings indicate that adding immunotherapy to adjuvant treatment does not significantly increase the incidence of adverse events.

A meta-analysis on postoperative adjuvant treatment of gastric cancer that included 11 randomized controlled clinical studies reported better improvement in OS rates in patients who received postoperative adjuvant combined immunotherapy than in those who received surgery alone (HR = 0.72; 95% CI = 0.61–0.85; *p* < 0.001) (28). In the GERCOR NEONIPIGA study, which included patients with locally defective mismatch repair/high microsatellite instability gastric or gastroesophageal junction adenocarcinoma, no recurrences or metastases were observed after neoadjuvant treatment followed by postoperative adjuvant immunotherapy until the reporting cutoff date (29). These findings indicate that in patients with locally advanced esophageal cancer after R0 resection, whether postoperative immunotherapy can achieve good therapeutic effects remains an important question. In the current study, both patient groups experienced varying degrees of local recurrence and distant metastasis within 2 years postoperatively. However, the incidence rates of local recurrence and distant metastasis were lower in the patients who received postoperative immunotherapy combined with chemotherapy than in those who received postoperative adjuvant chemotherapy alone, although the difference was not significant. In the CheckMate 577 trial, patients who underwent R0 resection after preoperative neoadjuvant chemoradiotherapy and received nivolumab as postoperative adjuvant treatment demonstrated superior DFS compared to those who received placebo (25). However, a report by Park et al. indicated that using durvalumab as maintenance therapy postoperatively did not provide superior DFS or OS compared to placebo (27). Thus, the role of postoperative immunotherapy in improving survival outcomes for resectable locally advanced esophageal cancer remains controversial. The PILOT trial aims to evaluate the efficacy of perioperative tislelizumab use, with hopes of achieving better 2-year DFS (30). Similarly, the multicenter phase III clinical trial NCT05495152 is further assessing the efficacy of postoperative immunotherapy in ESCC (31).

Neoadjuvant therapy combined with surgery is the standard treatment regimen for locally advanced esophageal malignancies. The efficacy of postoperative adjuvant therapy after R0 resection varies among different centers as the modalities used differ, with postoperative adjuvant chemotherapy being more frequently implemented in China. Early animal model studies suggest that neoadjuvant immunotherapy may be more effective than postoperative adjuvant treatments (32). In this study, patients in the aICT group did not achieve improved PFS compared to those in the aCT group. We posits that following primary tumor resection, the human body lacks specific antigens related to the primary tumor, resulting in ineffective postoperative immunotherapy. Therefore, whether postoperative adjuvant chemotherapy combined with immunotherapy can enhance antitumor efficacy and improve postoperative prognosis for esophageal cancer remains a matter of debate. As such, patients considering postoperative adjuvant immunotherapy must have a thorough understanding of relevant treatment information. This includes comprehensive knowledge of the treatment’s purpose, potential effects, and risks and side effects, as well as alternative treatment options. Furthermore, to ensure the ethical and legal integrity of the treatment process, oversight and review by an ethics committee is required.

Postoperative adjuvant chemotherapy typically consists of four cycles. Currently, the number of cycles for postoperative immunotherapy has not been defined. Based on studies in lung cancer, postoperative immunotherapy maintenance treatment is commonly administered for a duration of 1 year (33, 34); however, a real-world study by Kwak et al. indicated that many patients are unable to complete the postoperative immunotherapy maintenance treatment on schedule (35). Therefore, further clinical trials are needed to verify the appropriate number of cycles of postoperative immunotherapy in esophageal cancer. Given the lack of imaging techniques targeting the primary lesion as a main evaluation measure for effectiveness, Powles et al. reported the guiding role of ctDNA in adjuvant immunotherapy for urothelial carcinoma (36). It is essential to determine which patients will benefit from postoperative adjuvant immunotherapy for esophageal malignancies and to identify effective monitoring methods during the immunotherapy period of adjuvant treatment.

This study has some limitations. It was a single-center retrospective study with a small sample size, and the results may be influenced by specific factors related to the center’s environment, equipment, and personnel, thereby limiting their generalizability and applicability. Nevertheless, retrospective studies can still hold significant value in certain contexts. Future research should consider expanding the sample size and adopting a multicenter design to enhance the study’s representativeness and credibility.

In conclusion, based on our 2-year follow-up results, adjuvant chemotherapy combined with immunotherapy after R0 surgical resection does not provide superior DFS compared to postoperative chemotherapy alone for locally advanced esophageal malignancies.

## Data Availability

The original contributions presented in the study are included in the article/supplementary material, further inquiries can be directed to the corresponding authors.
